# Imipridone Anticancer Compounds Ectopically Activate the ClpP Protease and Represent a New Scaffold for Antibiotic Development

**DOI:** 10.1534/genetics.119.302851

**Published:** 2020-02-24

**Authors:** Samuel Jacques, Almer M. van der Sloot, Caroline C. Huard, Jasmin Coulombe-Huntington, Sarah Tsao, Sylvain Tollis, Thierry Bertomeu, Elizabeth J. Culp, Daniel Pallant, Michael A. Cook, Eric Bonneil, Pierre Thibault, Gerard D. Wright, Mike Tyers

**Affiliations:** * Institute for Research in Immunology and Cancer, University of Montréal, Quebec H3T 1JH, Canada; † David Braley Center for Antibiotic Discovery, McMaster University, Hamilton, Ontario L8S 4L8, Canada; ‡ Michael G. DeGroote Institute for Infectious Disease Research, McMaster University, Hamilton, Ontario L8S 4K1, Canada; § Department of Biochemistry and Biomedical Sciences, McMaster University, Hamilton, Ontario L8S 4K1, Canada

**Keywords:** AAA+ ATPase, antibiotic, CLPP, CRISPR screen, imipridone, MIPEP, mitochondrion, proteolysis, proteome, synergism

## Abstract

Systematic genetic interaction profiles can reveal the mechanisms-of-action of bioactive compounds. The imipridone ONC201, which is currently in cancer clinical trials, has been ascribed a variety of different targets. To investigate the genetic dependencies of imipridone action, we screened a genome-wide clustered regularly interspaced short palindromic repeats (CRISPR) knockout library in the presence of either ONC201 or its more potent analog ONC212. Loss of the mitochondrial matrix protease CLPP or the mitochondrial intermediate peptidase MIPEP conferred strong resistance to both compounds. Biochemical and surrogate genetic assays showed that impridones directly activate CLPP and that MIPEP is necessary for proteolytic maturation of CLPP into a catalytically competent form. Quantitative proteomic analysis of cells treated with ONC212 revealed degradation of many mitochondrial as well as nonmitochondrial proteins. Prompted by the conservation of ClpP from bacteria to humans, we found that the imipridones also activate ClpP from *Escherichia coli*, *Bacillus subtilis*, and *Staphylococcus aureus* in biochemical and genetic assays. ONC212 and acyldepsipeptide-4 (ADEP4), a known activator of bacterial ClpP, caused similar proteome-wide degradation profiles in *S. aureus*. ONC212 suppressed the proliferation of a number of Gram-positive (*S. aureus*, *B. subtilis*, and *Enterococcus faecium*) and Gram-negative species (*E. coli* and *Neisseria gonorrhoeae*). Moreover, ONC212 enhanced the ability of rifampin to eradicate antibiotic-tolerant *S. aureus* persister cells. These results reveal the genetic dependencies of imipridone action in human cells and identify the imipridone scaffold as a new entry point for antibiotic development.

## Abstract (precis)

The imipridones ONC201 and ONC212 selectively kill cancer cells and have been ascribed multiple mechanisms-of-action. Genome-wide CRISPR knockout screens revealed that loss of the mitochondrial proteases CLPP and MIPEP confer strong resistance to both compounds...

THE AAA+ ClpP protease complex has been extensively characterized as a major regulator of proteostasis in bacteria (Sauer and Baker 2011; Culp and Wright 2017) and to a lesser extent in the human mitochondrion (Quirós *et al.* 2015; Glynn 2017). ClpP peptidase complexes are composed of two components: a ClpP peptidase subunit and an ATP-dependent unfoldase subunit. Most organisms have only a single ClpP isoform (designated CLPP in humans), which operates with one or more Clp ATPases that include ClpX (CLPX in humans), ClpA, ClpC, or others depending on the species (Olivares *et al.* 2018). The ClpP subunits assemble in a barrel-shaped complex of two heptameric rings that form a spatially restricted proteolytic chamber with 14 internal catalytic cleavage sites. Access to the ClpP chamber is tightly regulated and requires a hexameric ATP-dependent unfoldase complex that physically caps the heptameric ClpP rings, and controls opening of the axial pores at one or both ends of the barrel (Olivares *et al.* 2018). The unfoldase complex mediates substrate recognition, protein unfolding, and translocation into the proteolytic chamber (Olivares *et al.* 2018). In the absence of the unfoldases, the axial pores of the ClpP peptidase barrel remain closed to prevent uncontrolled protein degradation (Olivares *et al.* 2018). Substrate recognition depends on specific linear sequence motifs located at either the N- or C-terminus of the substrate (Flynn *et al.* 2003; Sauer and Baker 2011). Bacteria also possess a cotranslational degradation tag system that attaches an 11-residue ssrA peptide to the C-termini of ribosomally stalled proteins to release and target the stalled protein for degradation by ClpXP (Sauer and Baker 2011). Bacterial ClpP and human mitochondrial CLPP are synthesized as inactive zymogens, and require N-terminal proteolytic processing to generate the mature proteolytically active forms (Maurizi *et al.* 1990; Kang *et al.* 2004).

ClpP is an attractive but complex antibiotic target. Although inhibition of the dual ClpP1/P2 system of *Mycobacterium tuberculosis* results in cell death (Famulla *et al.* 2016), in most bacterial species, ClpP is not essential (Culp and Wright 2017). ClpP is often important for virulence but since loss of ClpP can also confer antibiotic resistance, ClpP inhibition may not be a judicious general strategy (Culp and Wright 2017). In contrast to inhibition of ClpP, the acyldepsipeptide (ADEP) natural products kill bacteria by activating ClpP in an unregulated fashion in the absence of the Clp ATPases (Brötz-Oesterhelt *et al.* 2005). ADEPs directly bind the Clp ATPase docking site of the peptidase complex (Li *et al.* 2010) and stabilize the axial pore in an open position, thereby affording unrestricted access to the catalytic chamber (Olivares *et al.* 2018). ADEPs inhibit the growth of Gram-positive pathogens by causing massive unregulated intracellular proteolysis (Brötz-Oesterhelt *et al.* 2005), and when used in combination with other antibiotics can efficiently eradicate antibiotic-tolerant persister cells (Conlon *et al.* 2013). Even though ADEPs and various synthetic analogs demonstrate the potential of ClpP as a drug target (Bhandari *et al.* 2018; Wong and Houry 2019), the low solubility, chemical instability, and poor pharmacodynamics of ADEPs have hampered therapeutic development (Brötz-Oesterhelt *et al.* 2005; Moreno-Cinos *et al.* 2019).

In the mitochondrion, the CLPXP complex catalyzes the degradation of misfolded proteins, respiratory chain complex subunits, and other mitochondrial enzymes (Zhao *et al.* 2002; Quirós *et al.* 2015; Seo *et al.* 2016). An N-terminal targeting sequence directs the translocation of CLPP into the mitochondrial matrix, after which a series of poorly characterized proteolytic processing steps convert the CLPP proprotein into the mature active form (Bross *et al.* 1995). Accumulation of unfolded proteins in the mitochondrial matrix results in increased CLPP/CLPX expression and is an integral part of the mitochondrial unfolded protein response pathway (Zhao *et al.* 2002; Haynes *et al.* 2007). Disruption of CLPP or CLPX expression by knockdown impairs oxidative phosphorylation and ATP production, resulting in oxidative stress (Cole *et al.* 2015; Seo *et al.* 2016). Cancer cells appear to be more reliant on CLPP activity than normal cells (Seo *et al.* 2016). For example, CLPP is often overexpressed in acute myeloid leukemia (AML) and its inhibition induces death in AML cells, but not in normal hematopoietic cells (Cole *et al.* 2015; Bhandari *et al.* 2018). Pharmacological activation of mitochondrial CLPP by ADEP analogs can efficiently kill certain types of cancer cells (Wong *et al.* 2018).

The imipridone ONC201, originally known as TRAIL-inducing compound 10 (TIC10), was discovered in a forward chemical screen for p53-independent cytotoxic activity against a panel of cancer cell lines (Allen *et al.* 2013). The robust cancer-selective activity of ONC201 has led to its evaluation in > 20 phase II clinical trials for hematological malignancies and solid tumors (Allen *et al.* 2016). Several mechanisms of action have been ascribed to ONC201 and its more potent derivate ONC212 (Wagner *et al.* 2017), including: FOXO3A-mediated induction of TRAIL (Allen *et al.* 2013) upon inhibition of the prosurvival AKT/ERK pathway (Allen *et al.* 2013); activation of the integrated stress response (ISR) via the eIF2α kinases HRI and PKR, resulting in ATF4/CHOP-mediated upregulation of the TRAIL DR5 receptor (Kline *et al.* 2016); activation of the ISR independent of eIF2α (Ishizawa *et al.* 2016); and antagonism of dopaminergic G protein-coupled receptors (GPCRs) or activation of other GPCRs (Kline *et al.* 2018; Madhukar *et al.* 2019; Nii *et al.* 2019; Prabhu *et al.* 2019). Cell biological characterization suggests that ONC201 disrupts mitochondrial function and that cancer cells that do not depend on mitochondrial respiration are ONC201-insensitive (Greer *et al.* 2018). It has recently been shown that ONC201 and other imipridones directly bind and activate CLPP in the absence of CLPX, resulting in the degradation of selected mitochondrial proteins and loss of mitochondrial integrity (Graves *et al.* 2019; Ishizawa *et al.* 2019).

In this study, we used unbiased clustered regularly interspaced short palindromic repeats (CRISPR)-based loss-of-function genetic screens in the human NALM-6 pre-B cell lymphocytic cell line (Bertomeu *et al.* 2018) to identify the targets of ONC201 and ONC212. These screens recovered CLPP and the mitochondrial intermediate peptidase MIPEP as the top resistance hits. We confirm that ONC201 and ONC212 directly activate CLPP *in vitro*, demonstrate that MIPEP is required for processing the pro-CLPP zymogen into its active form, and show that mitochondrial proteins are the predominant targets of unregulated CLPP. We demonstrate that ONC212 is a potent activator of bacterial ClpP with antibiotic activity against multiple species, and that ONC212 synergistically enhances the activity of known antibiotics against *Staphylococcus aureus*, including drug-tolerant persister cells in biofilms. The favorable chemical and pharmacodynamic properties of the imipridones suggest these compounds might be developed as a new antibiotic scaffold to help combat the antimicrobial resistance crisis (Tyers and Wright 2019).

## Materials and Methods

### Human cell culture and genetic manipulation

Cells were grown in RPMI-1640 (NALM-6) or Dulbecco’s Modified Eagle Medium (HEK293T and RPE1-hTert) supplemented with 10% fetal bovine serum. For growth inhibition curves, parental or knockdown/knockout cell lines were grown in 384-well plates for 3 days, and viability assessed by CellTiter-Glo assay (Promega, Madison, WI). Statistical analysis was performed using Activity Base software (IDBS). For knockdown cell populations, short hairpin RNAs (shRNAs) against CLPP messenger RNA were cloned in a lentiviral pLKO.1-puro vector obtained from the Institute for Research in Immunology and Cancer (IRIC) High-Throughput Screening platform. Lentiviral particles were added to NALM-6 and RPE1-hTert cells at an MOI of 0.5, incubated for 2 days, and selected with 0.5 µg/ml (NALM-6) or 3 µg/ml (RPE1-hTert) puromycin for 4 days. For knockout cell populations, single guide RNAs (sgRNAs) against CLPP, MIPEP, and control loci were cloned in the all-in-one lentiCRISPRv2GFP plasmid (Addgene #82416). Lentiviral particles were added to NALM-6 cells at an MOI of 0.7, incubated for 4 days, and GFP-positive cells were sorted on a BD FACSAria II.

### Genome-wide CRISPR knockout screens

A doxycycline-inducible Cas9 clone of NALM-6 was transduced with the extended knockout (EKO) sgRNA library as described (Bertomeu *et al.* 2018), induced with doxycycline (2 µg/ml) for 7 days, and then treated with either ONC212 (150 nM), ONC201 (10 µM), or DMSO for 8 more days. Genomic DNA was extracted using the Gentra Puregene Cell Kit (QIAGEN, Valencia, CA), sgRNA barcodes were amplified by two rounds of PCR, purified using solid-phase reversible immobilization beads (AxyPrep FragmentSelect-I Kit; Axygen), and sequenced on a NextSeq500 system (Illumina). sgRNA frequencies were scored with the RANKS algorithm (Bertomeu *et al.* 2018).

### Yeast strain construction and culture

Human and bacterial genes were codon- and GC-content optimized for expression in *Saccharomyces cerevisiae* and synthesized *de novo*. *S. cerevisiae* strains expressing human CLPP and bacterial ClpP from the *GAL1* promoter were grown in synthetic complete (SC) dropout medium at 30° with continuous shaking, and monitored at OD_595_ at 15-min intervals in an automated reader (Tecan Sunrise). Apparent MICs were calculated as relative cell growth for drug *vs.* media only. Data were plotted using GraphPad Prism software. For disk diffusion assays, log-phase cultures were spread on selective 1% raffinose/1% galactose plates and incubated with drug-impregnated filter disks at 30° for 48 hr.

### Bacterial culture

MIC determinations were according to the Clinical and Laboratory Standards Institute (CLSI) guidelines (CLSI 2015). For disk assays, *S. aureus* or *Escherichia coli* were grown in LB medium overnight at 37°, spread on LB agar, and incubated at 37° for 24 hr. For synergy assays, *S. aureus* was cultivated in LB medium at 37° overnight then diluted 1/200 in fresh medium, and incubated with drug combinations in transparent 96-well flat-bottom plate wells and incubated for 24 hr at 37° prior to OD_600_ determination on a microplate reader (Tecan Infinite M1000 Pro). *S. aureus* biofilms were generated in 96-well plates as described for 48 hr in brain heart infusion (BHI) medium (Conlon *et al.* 2013), treated with drugs, and incubated for 72 hr at 37°. After aspiration, biofilm cells were dislodged by sonication and viable cells determined as described (Hazan *et al.* 2012), based on interpolation or extrapolation from a standard curve.

### Protein analysis and enzyme assays

Immunoblotting of human and yeast protein extracts was performed with mouse monoclonal anti-CLPP (Origene), rabbit polyclonal anti-MIPEP (GeneTex), mouse monoclonal anti-PGK1 (Abcam), and rabbit monoclonal anti-GAPDH (Cell Signaling Technologies) antibodies. HRP-conjugated goat anti-rabbit or goat anti-mouse (Jackson ImmunoResearch Laboratories) secondary antibodies were detected with Ultrascence Western Substrate (FroggaBio) and imaged on a ChemiDoc MP (Bio-Rad, Hercules, CA). *In vitro* degradation assays were performed with purified recombinant human CLPP (Profoldin) or *E. coli* ClpP (Li *et al.* 2010) at 37° in 384-well plates in triplicate using fluorescein isothiocyanate (FITC)-labeled casein (FITC-casein) or N-acetyl-Trp-Leu-Ala-7-amino-4-methylcoumarin (Ac-WLA-AMC) substrates (Graves *et al.* 2019). Fluorescence intensity was measured at λ_ex_/λ_em_ of 485/528 or 350/460 nm for FITC-casein and Ac-WLA-AMC, respectively. For gel-based assays, recombinant purified CLPP or ClpP was incubated with bovine α-casein at 30°, followed by 12% SDS-PAGE and protein detection by Coomassie Brilliant Blue.

### Mass spectrometry

NALM-6 cell ClpP knockdown or control populations in biological replicates were treated with either 150 nM ONC212 or DMSO vehicle for 24 hr, and harvested by centrifugation. Protein pellets were alkylated, digested with trypsin, and separated on a reversed-phase column on an Easy-NLC 1000 connected to a Thermo Fisher Scientific Orbitrap Fusion equipped with high-field asymmetric waveform ion mobility spectrometry (FAIMS) Pro (Pfammatter *et al.* 2018). Mass spectrometry (MS) spectra acquired at a resolution of 120,000 were followed by MS-MS analyses on the most abundant multiply charged precursor ions by collision-induced dissociation (CID) and acquired in the ion trap. FAIMS compensation voltages (CVs) were stepped from −37 to −93 V. Data were processed with PEAKS X (Bioinformatics Solutions) with standard search criteria and visualized with Scaffold 4.3.0. Stationary-phase *S. aureus* cells in biological replicate were treated with 30 μM ONC212 for 10 min, 40 min, and 24 hr at 37°. Cell pellets were lysed in 8 M urea and cleared by centrifugation. Next, 200 μg of total protein was alkylated, digested with trypsin, and separated on a C18 column and analyzed as above (Pfammatter *et al.* 2018). Data were searched against a custom *S. aureus* ATCC29213 database constructed from the draft genome sequence (Soni *et al.* 2015).

### Bioinformatics

Gene ontology term enrichment was calculated using custom scripts to determine *P*-values (Fisher’s exact test) and false discovery rate values (The Gene Ontology Consortium 2019). Mitochondrial genes were defined by gene ontology terms. For *S. aureus*, gene ontology annotations were drawn from UniProt for the C0673 strain. Orthologous genes for the *S. aureus* strain used in this study (ATCC 29213) were generated by sequence alignment to C0673.

### Data availability

Strains and plasmids are available on request. Supplemental Material, Table S1 contains CRISPR screen data for NALM-6 cells treated with ONC201 and ONC212; Table S2 contains quantitative proteomic scores for NALM-6 cells treated with ONC212; Table S3 contains quantitative proteomic scores for *S. aureus* cells treated with ONC212; Table S4 contains oligonucleotide primer sequences; Table S5 contains plasmids and DNA sequences; Table S6 lists *S. cerevisiae* strains; and Table S7 lists bacterial species and strains. CRISPR screen sequencing data have been deposited at the National Center for Biotechnology Information Gene Expression Omnibus under accession number GSE138894. Mass spectrometry data have been deposited with the ProteomeXchange Consortium via the Proteomics Identification Database partner repository (Perez-Riverol *et al.* 2019) with data set identifier PXD016119 (DOI: 10.6019/PXD016119).

Additional details on materials and methods used in this study are provided in the Supplemental material. Supplemental material available at figshare: https://doi.org/10.25386/genetics.11873841.

## Results

### Loss of CLPP and MIPEP confers resistance to ONC201 and ONC212

**Figure 1:**
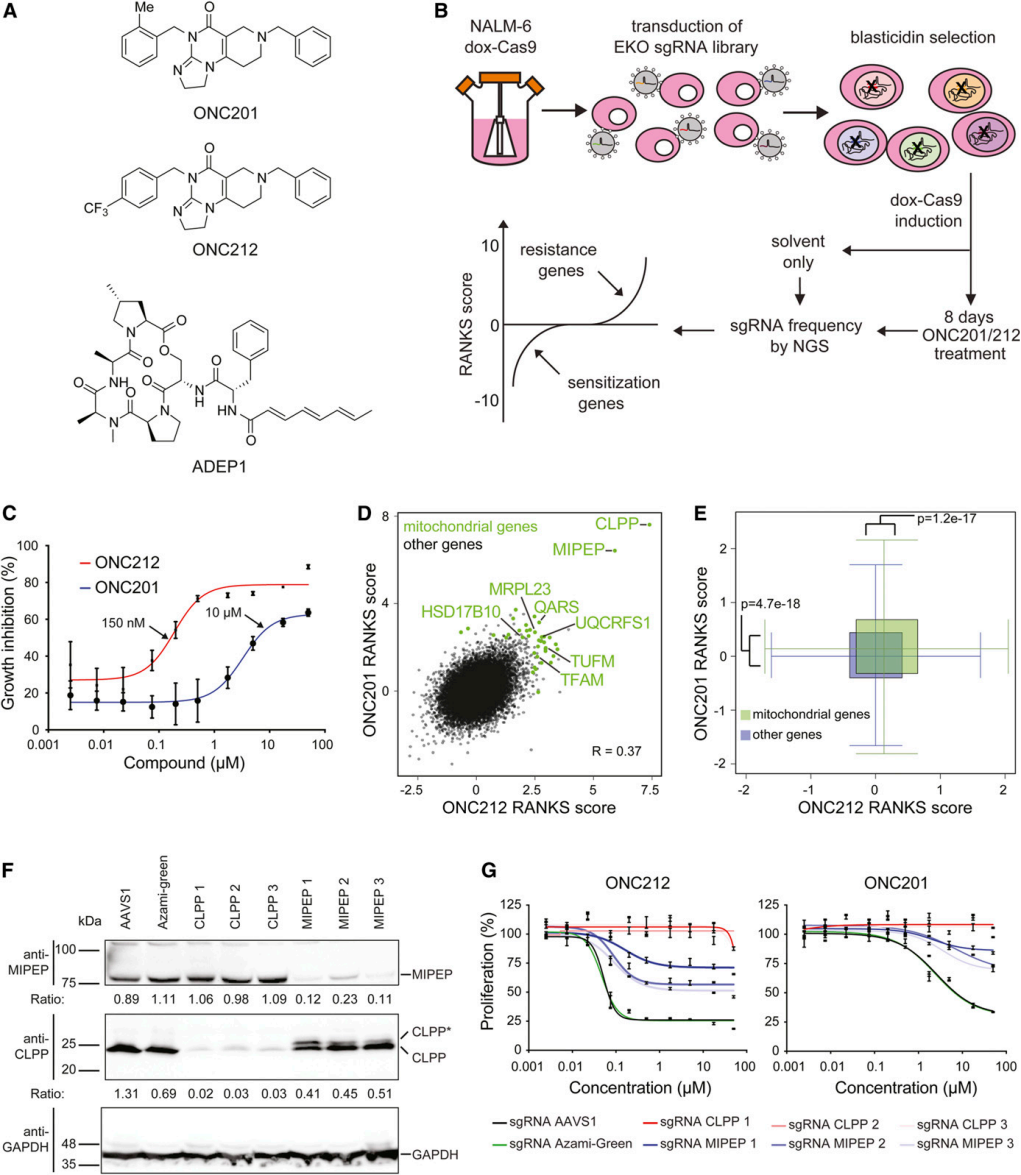
Genome-wide CRISPR screens identify CLPP as the target of ONC201 and ONC212. (A) Chemical structures of ONC201, ONC212, and ADEP1. (B) Schematic of CRISPR screen workflow. (C) Dose–response curves for ONC201 and ONC212 effects on NALM-6 cell proliferation, as assessed by bioluminescent quantitation of cellular ATP levels after 72-hr growth in the indicated drug concentrations. Values normalized to 0.1% DMSO controls are shown as mean ± SD (*n* = 4). Arrows indicate ONC201 and ONC212 concentrations used for screens. (D) Correlation between ONC201/ONC212 screens. The RANKS algorithm was used to calculate gene-level scores for each screen normalized to an untreated library pool. Mitochondrial genes that passed the false discovery rate threshold in either screen are indicated in green. (E) Enrichment of mitochondrial gene scores compared to all other genes in ONC201/212 screens. Whiskers extend to 95% C.I.s. (F) Immunoblot analysis of CLPP, MIPEP, and GAPDH protein in NALM-6 cell line knockout populations for indicated control (AAVS1 or Azami-Green), CLPP, and MIPEP sgRNAs cloned in a lentiviral vector that also expressed Cas9. Analysis was carried out on populations sorted for GFP-positive cells 4 days post-transduction and grown for a further 6 days. CLPP and MIPEP ratios were determined by image analysis, and normalized to the average of AAVS1 and Azami-Green controls. CLPP* denotes partially processed CLPP. (G) Proliferation of NALM-6 cell line knockout populations treated with ONC201 or ONC212. Populations generated as in (A) were assessed by bioluminescent quantitation of cellular ATP levels after 72-hr growth in the indicated drug concentrations. Values normalized to 0.1% DMSO controls are shown as mean ± SD (*n* = 8, in two separate duplicates). CRISPR, clustered regularly interspaced short palindromic repeats; dox, doxycycline; sgRNA, single guide RNA.

Systematic genetic interaction profiles, as detected by growth of a genome-wide loss-of-function pool in the presence of a drug or other bioactive compound, can often yield insights into a compound’s mechanism of action (Ho *et al.* 2011). In particular, genetic resistance to drug action can identify drug targets or downstream effectors in human cells (Lin *et al.* 2019). To systematically interrogate the mechanism of action of the imipridones ONC201 and ONC212 (Figure 1A), we carried out a genome-wide CRISPR knockout screen in the NALM-6 pre-B cell lymphocytic leukemia cell line in the presence or absence of each compound (Figure 1B). We used the previously described EKO library composed of 278,754 sgRNAs that target 19,084 Reference Sequence genes, 20,852 alternatively spliced exons, and 3872 hypothetical genes (Bertomeu *et al.* 2018). Dose–response curves of NALM-6 in the presence of ONC201 or ONC212 were generated to determine compound concentrations that yielded ∼50% growth inhibition (IC_50_), to allow detection of gene knockouts that cause sensitivity or resistance to each compound. As for other cell lines (Wagner *et al.* 2017), ONC212 exhibited more potent activity than ONC201 in NALM-6 cells, with IC_50_ values of 0.2 μM *vs.* 3.3 μM, respectively (Figure 1C). Frequencies of sgRNA abundances before and after treatment were normalized to DMSO controls, and converted to gene-level scores by the RANKS algorithm (Bertomeu *et al.* 2018). The significant overlap in the set of top-scoring genes between the two profiles (*R* = 0.37) was consistent with a similar mechanism of action for each compound. Both screens identified the same two strong resistance hits in the mitochondrial proteases CLPP and MIPEP (Figure 1D and Table S1). Many other genes encoding mitochondrial proteins also scored as resistance hits, including protein translation factors (TUFM, GFM1, and C12orf65), import receptor subunits (TOMM20 and TOMM22), ribosomal subunits and assembly factors (MRPS5, MRPS6, MRPS33, MRPL3, MRPL21, and MRPL23), electron transport chain components (UQCRFS1, UQCRC1, NDUFA2, and CYCS), transfer RNA (tRNA) synthetases (QARS, DARS2, EARS2, and SARS2), transcription factor A (TFAM), and ribonuclease P subunits (HSD17B10 and KIAA0391/MRPP3). Statistical enrichment analysis confirmed that mitochondrial genes had significantly higher resistance scores on average compared to all other genes in both screens (Figure 1E). None of the other previously suggested targets of ONC201 or ONC212 were found to be significantly depleted or enriched in the NALM-6 CRISPR screens, including TRAIL (TNFSF10), death receptor 5 (TNFRSF10B), dopamine receptor D2 (DRD2), the eIF2α kinases (HRI, PKR, PERK, and GCN2; also known as EIF2AK1-4, respectively), and GPCR 132 (GPR132) (Table S1). It remains possible that these other candidate targets are targets of the imipridones in other cell types. Overall, our genetic screens suggested that the mitochondrial proteases CLPP and MIPEP, as well as mitochondrial functions that may indirectly impact their activities, mediate the effects of ONC201 and ONC212.

### ONC201 and ONC212 activate CLPP

We validated CLPP and MIPEP using CRISPR-mediated insertion/deletion formation with three different sgRNAs for each gene, as well as control sgRNAs directed against AAVS1 and Azami-Green sequences. Detection with antibodies against CLPP or MIPEP confirmed protein loss in each of the knockout populations (Figure 1F). We detected a minor higher-molecular-weight species above the mature CLPP band only in the MIPEP knockout population (labeled CLPP*), which we suspected was due to a CLPP precursor-processing defect (see below). Loss of CLPP rendered cells completely resistant toward ONC201 and ONC212 treatment, whereas cells targeted with control sgRNAs remained sensitive, with IC_50_ values of 2.5 μM for ONC201 and 0.05 μM for ONC212 (Figure 1G). Similar resistance effects were observed upon shRNA-mediated knockdown of CLPP in NALM-6 cells, as well as in the primary retinal epithelial RPE1-hTert cell line (Figure S1, A and B). MIPEP sgRNAs rendered the cell population only partially resistant to ONC201/212 treatment (Figure 1G), likely because the knockout efficiency was lower than for CLPP (Figure 1F).

As the loss of MIPEP appeared to alter CLPP processing and thereby reduce CLPP activity, we reasoned that CLPP might be the direct target of ONC201/212. By analogy to the ADEP activation mechanism, we surmised that ONC201/212 might activate CLPP in the absence of its AAA + ATPase regulatory subunits. Thus, we assessed the effects of ONC212 on the enzymatic activity of purified recombinant mature CLPP *in vitro*. Full-length α-casein was completely degraded by CLPP in the presence but not absence of ONC212 (Figure S1C). We confirmed this result with *in vitro* CLPP assays that monitored the degradation of either fluorogenic peptide substrate (Ac-WLA-AMC) or full-length α-casein labeled with FITC in the presence of ONC201 or ONC212 (Figure S1D). Compared to ADEP1, a known activator of bacterial ClpP and human CLPP (Brötz-Oesterhelt *et al.* 2005; Wong *et al.* 2018), the imipridones were at least 20-fold more potent in these *in vitro* assays (Figure S1D). These results demonstrate that ONC201 and ONC212 inhibit human cell proliferation by inducing unregulated CLPP proteolytic activity, as recently shown in other studies (Graves *et al.* 2019; Ishizawa *et al.* 2019).

### Processing of CLPP by MIPEP

The presence of two CLPP species in MIPEP knockout populations suggested that MIPEP may process CLPP into a mature active form, but this interpretation was complicated by incomplete knockout of MIPEP at the population level (Figure 1F). Therefore, we selected control, CLPP, and MIPEP populations in the presence of ONC212 at the IC_50_ value for 3 days prior to analysis to enrich for cells resistant to the drug. Populations transduced with each MIPEP sgRNA had near complete loss of MIPEP and a strong enrichment for the putative CLPP precursor species when compared to control sgRNAs (Figure S2A). We also observed that ONC212 selection caused a reduction of MIPEP in control cultures, but the residual levels of MIPEP were sufficient to allow complete processing of CLPP. Since MIPEP levels in CLPP knockout cell populations cells were unaffected, cells may downregulate MIPEP as an adaptive response to ONC212, possibly because active CLPP degrades MIPEP in a negative feedback loop. These results strongly suggested that the primary effect of imipridone treatment depends on CLPP and that MIPEP is required for CLPP maturation.

**Figure 2:**
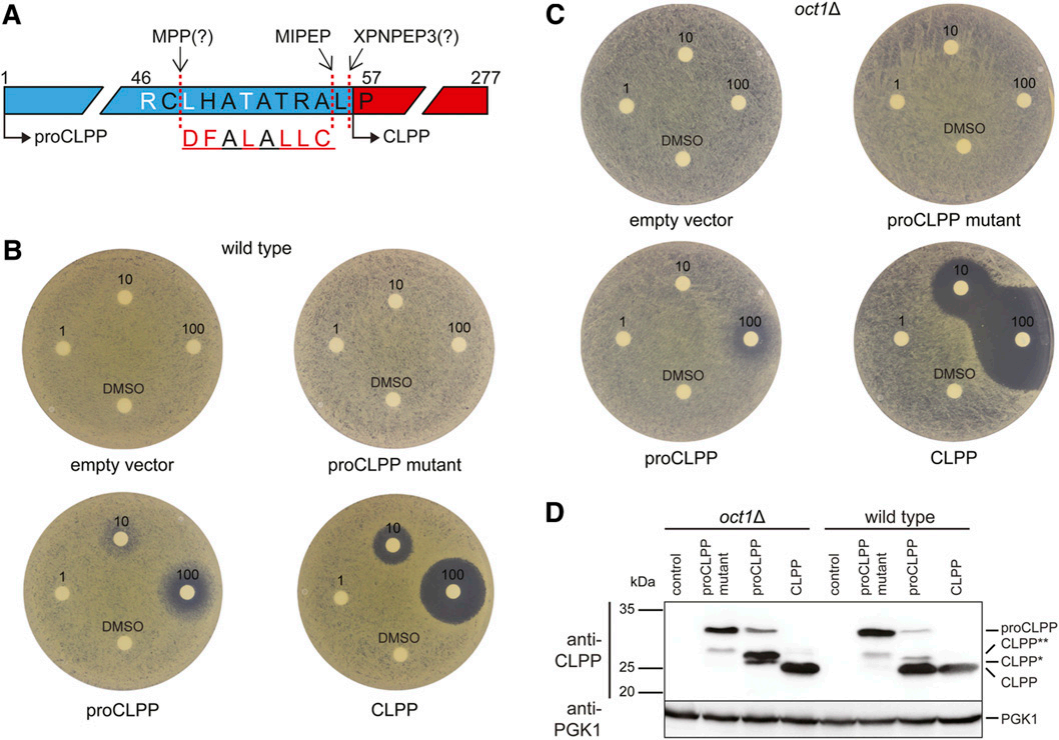
MIPEP processes precursor CLPP into mature CLPP. (A) Predicted processing motifs and cleavage sites in the N-terminal region of CLPP. Putative cleavage sites for MIPEP, MPP, and XPNPEP3 are indicated by arrows. The MPP/MIPEP R-10 recognition motif has the pattern: R-X↓(F/L/I)-X-X-(T/S/G)-X-X-X-X↓(Gakh *et al.* 2002). Following initial cleavage by MPP, the underlined octapeptide is cleaved by MIPEP and the remaining N-terminal Leu is removed by XPNPEP3 or another aminopeptidase (Singh *et al.* 2017). Scrambled MIPEP recognition motif in mutated proCLPP is shown below. (B) Disk diffusion assay of wild-type *S. cerevisiae* strains bearing either an empty vector or mature human CLPP, proCLPP, or a proCLPP mutant with a scrambled MIPEP recognition site, all expressed from the *GAL1* promoter. Mature CLPP was expressed with a Met substitution at position 56 to allow translation initiation. Disks contained either 0, 1, 10, or 100 nmol of ONC212. SC-His medium plates containing 2% galactose were incubated at 30° for 2 days. (C) As for (B) except in an *oct1*Δ mutant strain. (D) Immunoblot detection of human CLPP precursor isoforms produced in wild-type *S. cerevisiae* or an *oct1*Δ deletion mutant strain. Cultures of wild-type or *oct1*Δ strains were grown in liquid SC-His medium containing 2% galactose to midlog phase. Detection of yeast Pgk1 was used as loading control. Proposed processing events for CLPP are described in Figure S2D.

To investigate this hypothesis in a more genetically tractable system, we developed a surrogate genetic assay for human CLPP and MIPEP function in the budding yeast *S. cerevisiae*. Budding yeast lacks an endogenous CLPP ortholog but nevertheless bears orthologs of CLPX and MIPEP, encoded by *MCX1* and *OCT1*, respectively. Sequence alignments of CLPP orthologs were used to predict potential proteolytic processing sites needed for CLPP maturation (Figure 2A). We introduced plasmids that conditionally expressed mature human CLPP (residues 57–277), full-length human proCLPP (residues 1–277), and a proCLPP mutant in which a putative MIPEP recognition site was mutated into wild-type and *oct1*Δ strains (Figure 2, B and C). Expression of mature human CLPP and its precursor variants from the *GAL1* promoter was well tolerated as each strain grew as well as the empty vector control. This result suggested that yeast Mcx1 does not activate human CLPP under the growth conditions tested. Strikingly, in halo assays, ONC212 caused dose-dependent toxicity in strains that expressed either CLPP or proCLPP, but not the proCLPP mutant allele or empty vector (Figure 2B). ONC212 was 10-fold more toxic in an efflux pump-deficient yeast strain that expressed either CLPP or proCLPP (Figure S2, B and C). In the *oct1*Δ strain, which lacks the yeast ortholog of MIPEP, expression of the wild-type proCLPP allele conferred less sensitivity to ONC212 than in a wild-type strain (Figure 2C). The *oct1*Δ strain was even more sensitive to expression of mature CLPP in the presence of ONC212 than a wild-type strain for unknown reasons. Immunoblot analysis of CLPP revealed only a single major species for expression of mature CLPP in both the wild-type and *oct1*Δ strains (Figure 2D). Expression of full-length proCLPP yielded mainly mature CLPP in the wild-type strain but predominantly unprocessed forms of CLPP in the *oct1*Δ strain (labeled CLPP*, CLPP**, and proCLPP, the latter corresponding to the predicted size of full-length unprocessed proCLPP). The intermediate CLPP* and CLPP** isoforms were suppressed in the proCLPP mutant, suggesting that upstream processing events also depend on the MIPEP consensus motif (see Figure S2D for a detailed description). Co-electrophoresis of yeast and human cell extracts demonstrated that the CLPP mature isoform in yeast was equivalent in size to the mature form in NALM-6 cells, while the smallest unprocessed CLPP* isoform in yeast co-migrated with the unprocessed form in the MIPEP knockout NALM-6 cell line (see Figure S2E for details). Taken together, these results demonstrate that human proCLPP is processed by the yeast ortholog of MIPEP and that this processing is required for full activation of CLPP by ONC212. We note that the genetic dependency scores for CLPP and MIPEP are closely correlated across hundreds of genome-wide CRISPR screens in different cell lines (Meyers *et al.* 2017), consistent with the requirement for MIPEP in CLPP maturation (Figure S2F).

### Effect of CLPP activation on the human proteome

**Figure 3:**
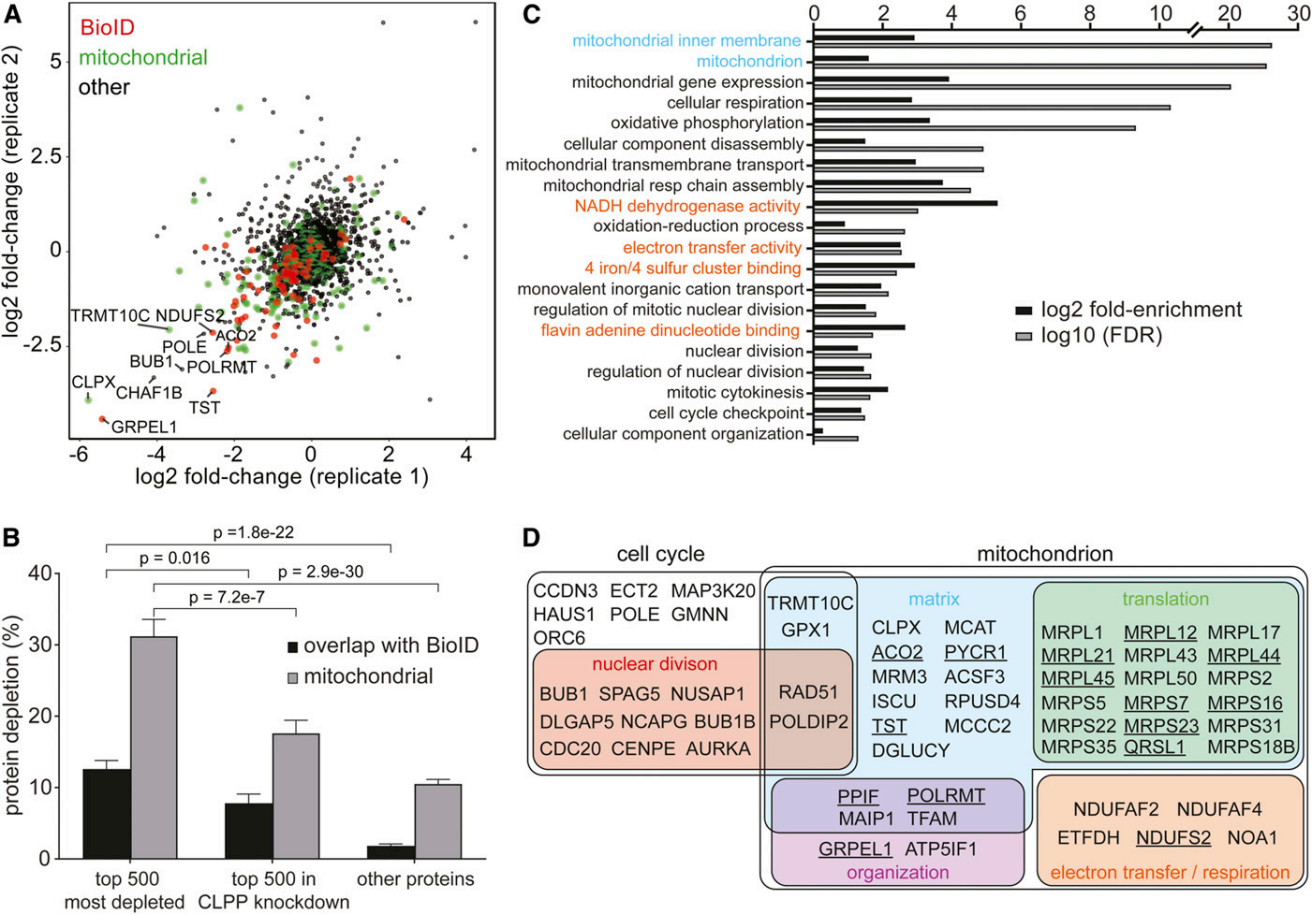
Effect of ONC212 treatment on the human proteome. (A) Protein abundance changes in NALM-6 cells treated with 150 nM ONC212 compared to solvent-treated control cultures. Protein abundance was determined by label-free quantitative proteomics and expressed as log_2_ fold change of treated *vs.* untreated samples. Values are the average of biological duplicate experiments. Mitochondrial proteins are shown in green and all other proteins in black. CLPP-associated proteins previously identified by BioID proximity labeling (Ishizawa *et al.* 2019) are depicted in red. (B) Enrichment of CLPP-associated proteins identified by BioID (Ishizawa *et al.* 2019) and mitochondrial proteins in the top 500 depleted proteins after ONC212 treatment in wild-type *vs.* CLPP knockdown NALM-6 cells. (C) Gene ontology analysis of the top 500 depleted proteins in ONC212-treated cells. Mitochondrial-related gene ontology categories were significantly enriched (*P* = 2.9e−30, Fisher’s exact test). Cellular component (cyan), biological process (black), and molecular function (orange) terms are indicated. (D) Overrepresented locations and functional categories for proteins depleted upon ONC212 treatment. CLPP-associated proteins identified by BioID (Ishizawa *et al.* 2019) are underlined. FDR, false discovery rate.

We used unbiased label-free proteomics to determine changes in protein abundance upon imipridone treatment of human cells. A spectrum of interaction partners of mitochondrial CLPP have been previously identified by BioID proximity-labeling analysis (Cole *et al.* 2015; Ishizawa *et al.* 2019). In particular, ∼90 CLPP-associated proteins are depleted upon CLPP activation by either ONC201 treatment or a hyperactivating mutation (Ishizawa *et al.* 2019). To assess the proteome-wide effects of unregulated CLPP-mediated proteolysis in an unbiased fashion, NALM-6 cells expressing an shRNA directed against CLPP or a scrambled shRNA control were grown in duplicate in the presence or absence of ONC212, and analyzed by label-free quantitative proteomics using liquid chromatography (LC)-MS/MS combined with FAIMS to enhance proteome coverage (Pfammatter *et al.* 2018). Across all eight samples, 3375 proteins were detected with at least five unique peptides, and of these 342 showed at least twofold depletion upon ONC212 treatment (Figure 3A and Table S2). Correlations in protein abundance between biological replicates were within typical ranges for high-confidence protein identification at a proteome-wide scale (Geiger *et al.* 2012), with *R* > 0.72 and *R* > 0.95, for log2 abundance across ONC212-treated and -untreated replicates for all proteins detected and proteins with ≥ 10 unique peptides, respectively. Correlations for corresponding log2 fold change data were *R* = 0.24 for all 3375 proteins and *R* = 0.33 for 1979 proteins detected with ≥ 10 unique peptides. Of the top 500 most depleted proteins upon ONC212 treatment, 31% were known mitochondrial proteins and 13% were identified as CLPP-associated in a previous BioID study (Ishizawa *et al.* 2019). After shRNA-mediated CLPP knockdown, the fraction of mitochondrial proteins in the top 500 depleted proteins decreased to 18% and documented BioID partners to 8% (Figure 3B).

Gene ontology analysis of the top 500 depleted proteins in ONC212-treated cells revealed strong enrichment for mitochondrial-associated gene ontology terms (Figure 3, C and D). The most heavily depleted protein was GRPEL1, a nuclear exchange factor that is an essential component of the presequence translocase-associated motor (PAM) complex that mediates import of proteins into the mitochondrial matrix. Other depleted mitochondrial proteins included: ribosomal protein subunits (MRPS7 and MRPS22), subunit A of Glutamyl-tRNA (Gln) amidotransferase (QRSL1), a tRNA methyltransferase 10 homolog C (TRMT10C), subunits of the respiratory chain (NDUFS2, NDUFAF2, NDUFAF4, and ETFDH), RNA polymerase (POLRMT), metabolic enzymes (ACO2), and detoxifying enzymes (TST and GPX1). Some of the proteins we identified in our proteome-wide analysis have been previously shown to interact with the ClpXP complex under normal conditions by BioID proximity labeling (*e.g.*, ACO2, TST, POLDIP2, and TFAM) (Cole *et al.* 2015) or are substrates of ClpXP (*e.g.*, NOA1) (Al-Furoukh *et al.* 2014). While a few of the top depleted proteins overlapped with proteins identified by BioID analysis of activated CLPP, the vast majority did not (Figure 3D). Notably, the second most depleted protein, which was unique to our data set, was CLPX, the AAA+ ATPase regulatory subunit that directly binds and activates CLPP. This result suggests that CLPX may be degraded adventitiously by active CLPP and/or that CLPP activation may normally be self-limited by degradation of its physiologically obligate cofactor. CLPX also performs chaperone functions independently of CLPP, as it promotes heme biosynthesis and enhances the DNA-binding activity of TFAM, a mitochondrial transcription factor (Kasashima *et al.* 2012; Yien *et al.* 2017). TFAM itself was also depleted in response to ONC212, in agreement with previous studies in other cell lines (Greer *et al.* 2018; Graves *et al.* 2019). Consistent with previous reports of mitochondrial defects caused by ONC201 treatment in various cell lines (Greer *et al.* 2018; Ishizawa *et al.* 2019), we found that both ONC201 and ONC212, but not an inactive isomer of ONC201, caused a collapse of mitochondrial membrane potential and an increase in mitochondrial reactive oxygen species levels in NALM-6 cells (Figure S3). The broad impact of ectopic CLPP activation on the mitochondrial proteome indicates that the primary defect elicited by ONC212 is a collapse of mitochondrial integrity and function.

ONC212 treatment also induced the depletion of proteins implicated in cell division control and cytokinesis (Figure 3, C and D), including the mitotic Aurora kinase (AURKA), the activator of the G1/S transition cyclin D (CCND3), and an activator of the Anaphase Promoting Complex/Cyclosome (CDC20). At a lower detection threshold of three unique peptides, cyclins as a functional class were also significantly more depleted than other proteins (*P* = 0.00262; Mann–Whitney *U*-test). This unbiased proteomic analysis shows that ONC212-mediated activation of CLPP has a widespread destructive impact that extends beyond mitochondrial function.

### Imipridones activate bacterial ClpP from multiple species

**Figure 4:**
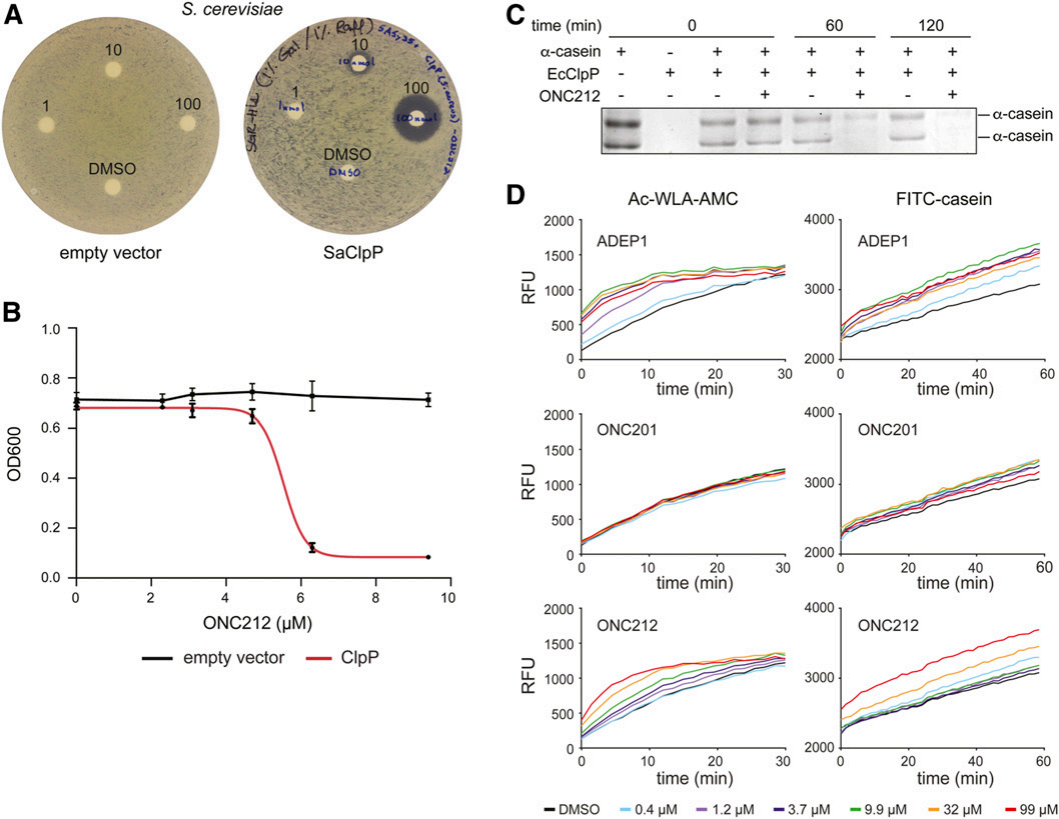
ONC201 and ONC212 activate bacterial ClpP (A). Disk diffusion assays on wild-type *S. cerevisiae* strains that either bear a plasmid expressing *S. aureus* ClpP (SaClpP) from the *GAL1* promoter or an empty vector. Disks contained 0, 1, 10, or 100 nmol ONC212. SC-His medium plates containing 2% galactose were incubated at 30° for 2 days (B). Growth curves for *S. cerevisiae* strains bearing either *S. aureus* ClpP expressed from the *GAL1* promoter or empty vector control grown in the presence of the indicated concentration of ONC212. OD_600_ measurements were taken after 48 hr. Values represent mean ± SD (*n* = 3). (C) Degradation of full-length unlabeled α-casein by purified recombinant *E. coli* ClpP in the presence of 100 µM ONC212 or 0.1% DMSO control. Proteins were resolved by SDS-PAGE and detected by Coomassie Brilliant Blue stain. (D) Effects of ADEP1, ONC201, and ONC212 on the degradation of fluorescent Ac-WLA-AMC peptide and FITC-casein substrates by purified recombinant *E. coli* ClpP. RFU values are the mean of triplicate measurements. RFU, relative fluorescent unit.

Given that ONC201 and ONC212 were more potent activators of CLPP than ADEP1, and the extensive conservation between mitochondrial CLPP and bacterial ClpP, we tested whether the imipridones can activate bacterial ClpP. We first used the yeast-based surrogate genetic assay to test for activation of *S. aureus* ClpP. As for human CLPP, expression of *S. aureus* ClpP did not impair yeast growth in the presence of a solvent control, whereas exposure of the strain to ONC212 in a halo assay caused a dose-dependent zone of growth inhibition (Figure 4A) and dose-dependent inhibition of growth in liquid culture with an IC_50_ of 5.4 μM (Figure 4B). We then assessed the ability of ONC212 to activate purified recombinant *E. coli* ClpP, which is known to be activated by ADEP1 (Brötz-Oesterhelt *et al.* 2005; Li *et al.* 2010). ONC212 potently induced *E. coli* ClpP catalytic activity *in vitro* as shown by the complete degradation of full-length α-casein (Figure 4C). ONC201, ONC212, and ADEP1 were then each tested for their ability to induce degradation of the fluorescent peptide Ac-WLA-AMC and FITC-casein. ONC201 had no effect on *E. coli* ClpP as fluorescence release from neither Ac-WLA-AMC or FITC-casein was detected above background hydrolysis rates. However, *E. coli* ClpP was activated by ONC212 for degradation of both the peptide and protein substrate, but only at ∼10-fold higher concentrations than the ADEP1 control (Figure 4D). These assays established that ONC212 was able to activate ClpP isoforms from both Gram-positive and Gram-negative species, and suggested that the imipridone scaffold might have antibiotic activity.

### ONC212 has ClpP-dependent antibacterial activity

**Figure 5:**
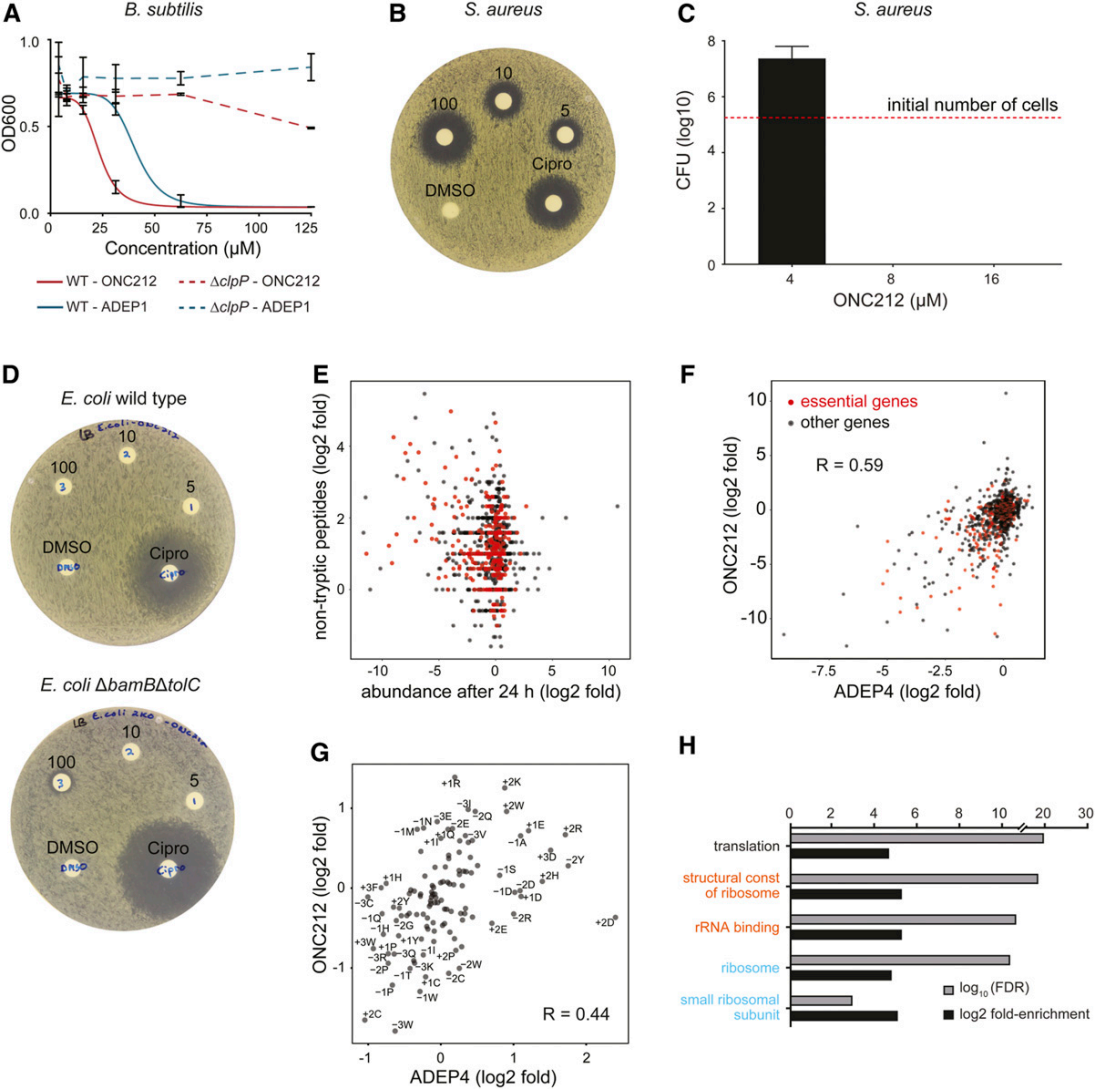
ClpP-dependent antibacterial activity of ONC212. (A) Growth of *B. subtilis* wild-type and Δ*clpP* strains in the presence of the indicated concentrations of ONC212 or ADEP1. Measurements were taken 24 hr after initial inoculation at OD_600_ = 5 × 10^−3^. Values represent mean ± SD (*n* = 3). (B) Disk diffusion assay on WT *S. aureus*. Disks contained either 0, 5, 10, or 100 nmol of ONC212 or 14 nmol cipro as positive control. Plates were incubated at 37° for 24 hr. (C) *S. aureus* was inoculated at a density of ∼1.9 x 106 cells/ml in the presence of 4, 8, or 16 µM ONC212 for 24 hr, then plated on nonselective medium to determine remaining CFUs. (D) Disk diffusion assay on WT *E. coli* or a Δ*bamB*Δ*tolC* hyperpermeable and efflux pump-deficient *E. coli* strain. Disks contained 5 (1), 10 (2), or 100 (3) nmol ONC212, DMSO solvent only as a negative control, or 2.7 nmol cipro as a positive control. (E) Quantitative mass spectrometric analysis of proteome-wide changes in *S. aureus* induced by 30 μM ONC212. Increase in nontryptic peptides, determined as log_2_ fold change of MS/MS spectra matched to nontryptic peptides between ONC212-treated and untreated samples (10- and 40-min samples combined), was plotted against log_2_ fold change in protein abundance, determined as total tryptic peptide precursor ion area, between ONC212-treated and untreated samples after 24 hr. Red indicates essential proteins (Chaudhuri *et al.* 2009). (F) Log_2_ fold change in protein abundance in ONC212-treated *vs.* untreated samples after 24 hr compared to published data for closest protein homologs in ADEP4-treated *S. aureus* (Conlon *et al.* 2013). Red indicates essential proteins. (G) Shifts in position-specific amino acid preferences around nontrypsin cleavage sites upon ONC212 *vs.* ADEP4 treatment. Log_2_ fold change in residue occurrence up to three residues away from protease cleavage site were calculated for ONC212 treatment and plotted against equivalent ratios for ADEP4 treatment (Conlon *et al.* 2013). (H) Gene ontology term analysis for protein depletion in ONC212-treated *S. aureus* cells. Cellular component (cyan), biological process (black), and molecular function (orange) terms are indicated. cipro, ciprofloxacin; FDR, false discovery rate; WT, wild-type.

We benchmarked the potential antimicrobial activity of ONC212 compared to an ADEP1 control. We first tested for activity against the Gram-positive species *Bacillus subtilis* in wild-type and isogenic Δ*clpP* deletion strains (Msadek *et al.* 1998) (Figure 5A). Growth inhibition was observed for both ONC212 and ADEP1 with IC_50_ values of 17 and 35 µM, respectively, a statistically significant difference (*P*-value = 0.01), whereas ONC201 did not impair *B. subtilis* growth (data not shown). Importantly, ONC212 and ADEP1 did not have any growth inhibitory effect in the *B. subtilis* Δ*clpP* strain, thereby demonstrating that ClpP is also the main target of ONC212 in bacteria. We then used disk diffusion assays and MIC determinations to assess the activity of the impridones and an ADEP1 control against *S. aureus*, a clinically important Gram-positive pathogen. *S. aureus* was highly sensitive to ONC212, which was around sixfold more potent than ADEP1, with respective MIC values of 8–16 μM *vs.* 50–100 μM (Figure 5B and Table 1). To determine whether ONC212 was bactericidal or bacteriostatic, *S. aureus* cultures were treated for 24 hr with three different ONC212 concentrations around the MIC value, washed to remove compound, plated on nonselective medium, and assessed for total numbers of colony-forming units (CFUs) before and after treatment. At an ONC212 concentration of 8 µM or higher, no cell growth after replating was observed demonstrating that ONC212 has bactericidal properties against *S. aureus* (Figure 5C). Finally, we assessed activity against *E. coli* as a representative Gram-negative species. A wild-type *E. coli* strain was resistant to both ONC201 and ONC212, as determined by disk diffusion assay (Figure 5D) and MIC determination (Table 2). Since Gram-negative bacteria have efficient efflux systems and an outer membrane that confer strong antibiotic resistance, we also tested ONC212 and ONC201 in a hyperpermeable and efflux-deficient *E. coli* strain, BW25113 Δ*bamB*Δ*tolC* (King *et al.* 2014). In this strain, ONC212 exhibited activity in a disk diffusion assay (Figure 5D and Figure S4) and a detectable MIC of 50–100 μM, whereas ONC201 exhibited only marginal activity (Table 2). Taken together, these results show that ONC212 has ClpP-dependent antimicrobial activity, particularly against Gram-positive species.

**Table 1:** MIC determinations for ONC201, ONC212, and ADEP1 against a panel of ESKAPE pathogens

Compound	*E. faecium* (ATCC 19434)	*S. aureus* (ATCC 29213)	*K. pneumonia* (ATCC 33495)	*A. baumannii* (ATCC 17978)	*P. aeruginosa* (PA01)	*E. aerogenes* (ATCC 13048)
ONC201 (μM)	250–500	250–500	> 500	> 500	> 500	> 500
ONC212 (μM)	62.5–125	8–16	> 250	> 250	> 250	> 250
ADEP1 (μM)	—	50–100	—	—	—	—

Strains are indicated in parentheses for each species. Dashes indicate not tested. ATCC, American Type Culture Collection.

**Table 2:** MIC determinations for ONC201, ONC212, and ADEP1 against a panel of non-ESKAPE bacteria or mycobacteria

Compound	*N. gonorrhoeae* (WHOY)	*N. gonorrhoeae* (ATCC 49226)	*M. smegmatis* (mc2155)	*M. tuberculosis* (H37Ra)	*E. coli* (BW25113)	*E. coli* (∆*tolC*∆*bamB*)
ONC201 (μM)	250–500	125–250	125–250	250–500	> 500	> 250
ONC212 (μM)	31.25–62.5	31.25–62.5	> 250	> 100	> 250	50–100
ADEP1 (μM)	—	—	> 360	—	—	—

Strains are indicated in parentheses for each species. Dashes indicate not tested. ATCC, American Type Culture Collection.

### Effect of ONC212-mediated ClpP activation on the bacterial proteome

To investigate the mechanism whereby ectopic ClpP activation kills bacteria cells, we carried out comparative proteomic analysis of ONC212-treated *vs.* -untreated cells. We used stationary-phase cultures of *S. aureus* to be able to directly compare our data to a previous proteome-wide analysis of the effects of ADEP4 (Conlon *et al.* 2013). Cultures were treated for 10 min, 40 min, and 24 hr with 30 μM ONC212 and analyzed by label-free quantitative proteomics on the LC-MS/MS-FAIMS platform described above (Table S3). This analysis revealed a large increase in nontryptic peptides in the ONC212-treated samples compared to untreated controls, consistent with an increase in ClpP-mediated proteolysis (Figure 5E). Proteins with an increased number of nontryptic peptides detected after 10 and 40 min of ONC212 treatment tended to be depleted after 24 hr of treatment (*R* = −0.18; *P* = 5.5e−8), indicating that ClpP-mediated degradation of many of the depleted proteins occurs as an immediate and direct effect of drug exposure. The 50 most depleted proteins after 24 hr of ONC212 treatment were enriched for essential genes (fold-enrichment = 1.76; *P* = 0.0032, Fisher’s exact test) (Chaudhuri *et al.* 2009). Comparison of the ONC212-treated proteome with the previous ADEP4-treated proteome (Conlon *et al.* 2013) revealed a strong correlation (*R* = 0.59) between the two data sets (Figure 5F), suggesting that ONC212 and ADEP4 have the same mode of action in cells. Of the 100 most depleted proteins in our data set with a homolog detected in the previous study (Conlon *et al.* 2013), 68 were also significantly depleted after ADEP4 treatment. We also found that ONC212 treatment induced shifts in position-specific amino acid preferences around nontryptic cleavage sites, similar to shifts induced by ADEP4 treatment (*R* = 0.44, *P* = 9.7e−7) (Conlon *et al.* 2013), again suggesting that the same protease is activated by both treatments (Figure 5G). The 200 most depleted proteins were enriched for ribosome-related functions (Figure 5H), also consistent with the previous analysis of ADEP4 effects on the proteome (Conlon *et al.* 2013).

### Assessment of ONC212 activity against other bacterial species

Given the attractive mode-of-action and antimicrobial activity profile of the imipridones against *S. aureus*, the sensitivity of other clinically relevant pathogenic bacteria to ONC201 and ONC212 was determined. In addition to *S. aureus*, we assessed other pathogens in the standard ESKAPE panel (*Enterococcus faecium*, *Klebsiella pneumoniae*, *Acinetobacter baumannii*, *Pseudomonas aeruginosa*, and *Enterobacter aerogenes*) (Rice 2008), *Neisseria gonorrhoeae*, and the *M. tuberculosis* surrogate strains *M. tuberculosis H37Ra* and *M. smegmatis* (Table 1 and Table 2). The Gram-positive species *E. faecium* was sensitive to ONC212 (MIC = 62.5–125 μM), whereas most Gram-negative species were insensitive toward ONC212 or ONC201. A notable exception was *N. gonorrhoeae*, which was sensitive to ONC212 (MIC = 31.25–62.5 μM), similar to the previously observed sensitivity of *Neisseria* species toward ADEP analogs (Goodreid *et al.* 2016). The mycobacteria *M. smegmatis* (MIC = 125–250 µM) and the attenuated *M. tuberculosis* H37Ra strain (MIC = 250–500 µM) were mildly sensitive to ONC201 but not ONC212, perhaps due to the more complex ClpP1/ClpP2 subunit structures in these species (Table 2). This survey indicated that Gram-positive pathogens are sensitive to ONC212, most Gram-negative pathogens with the exception of *N. gonorrhoeae* are resistant to ONC212, and that mycobacteria are somewhat sensitive toward ONC201 rather than ONC212.

### Synergism between ONC212 and other antibiotics

**Figure 6:**
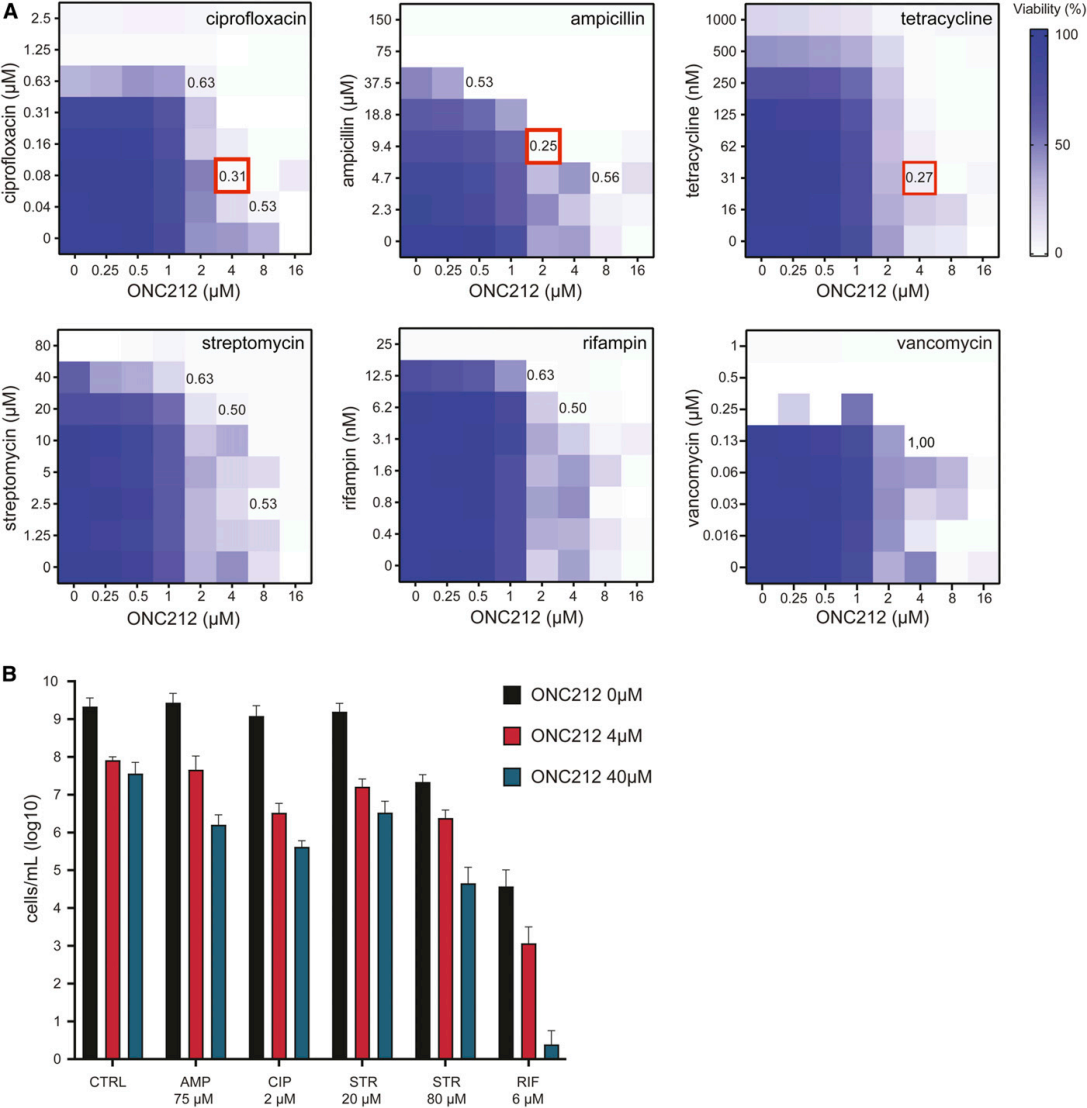
Effect of ONC212 in combination with different antibiotics on *S. aureus* growth. (A) The indicated antibiotics were tested in combination with ONC212 at the indicated concentrations. Each square within a checkerboard indicates cell growth determined as OD_600_ after 24 hr. FICI values indicated for particular concentrations. Red indicates a synergistic combination. (B) Preformed *S. aureus* biofilms were treated with indicated antibiotics alone or in combination with either 4 or 40 µM ONC212 for 72 hr. Biofilms were dislodged and surviving cells determined by OD_600_ values after culture in antibiotic-free medium for 16 hr. AMP, ampicillin; CIP, ciprofloxacin; CTRL, no-antibiotic control; FICI, fractional inhibitory concentration index; RIF, rifampin; STR, streptomycin.

Given that ADEP4 has previously been shown to synergize with other antibiotics (Conlon *et al.* 2013), we investigated potential synergism of ONC212 with six standard antibiotics of different mechanistic classes known to inhibit *S. aureus* growth: ciprofloxacin, ampicillin, tetracycline, streptomycin, vancomycin, and rifampin. Log-phase *S. aureus* cultures in rich LB medium were cotreated with pairwise combinations of ONC212 and each antibiotic across a range of concentrations. The fractional inhibitory concentration index (FICI) was used to determine synergism based on the standard criterion that FICI values of < 0.5 indicate synergism (Meletiadis *et al.* 2010; Tyers and Wright 2019). Combinations of ONC212 with ampicillin (2 µM ONC212 and 9.38 µM ampicillin), tetracycline (4 µM ONC212 and 31.25 nM tetracycline), or ciprofloxacin (4 µM ONC212 and 80 nM ciprofloxacin) each exhibited synergistic interactions with FICI values of 0.25, 0.27, and 0.31, respectively (Figure 6A). Combinations of ONC212 with either streptomycin or rifampin had FICI values of 0.5 that indicated additivity, while the combination with vancomycin failed to show any interaction with a FICI value of 1.00 (Figure 6A). These results demonstrate the potential for ONC212 to augment the activity of established antibiotics.

### ONC212 and rifampin combine to eliminate biofilm formation

Persister cells are phenotypically dormant pathogenic bacteria that are often encountered in chronic infections and difficult to eliminate due to their high antibiotic tolerance (Lewis 2005). A combination of ADEP4 and rifampin at clinically achievable concentrations has been shown to eradicate *S. aureus* biofilms in both *in vitro* and *in vivo* infection models (Conlon *et al.* 2013). We tested the ability of ONC212 alone and in combination with various antibiotics to eliminate *S. aureus* biofilms in 96-well plate format. Established biofilms were treated with antibiotics for 72 hr and, after washing away antibiotics, dislodged cells were cultured in antibiotic-free medium to determine CFUs. ONC212 alone at 4 or 40 µM showed only modest activity against persister cells in the biofilm. Most of the other antibiotics alone or in combination with ONC212 at 4 or 40 µM also showed only modest reductions in the number of surviving cells (Figure 6B). Treatment combinations that were synergistic when tested against log-phase *S. aureus* cultures (*i.e.*, ampicillin and ciprofloxacin, see above) were essentially inactive against biofilm persister cells. However, a combination of low-dose rifampin (6.3 µM) with ONC212 at 40 µM caused a 10^4^-fold reduction in surviving persisters (Figure 6B). This result suggests that imipridone analogs might be efficacious in combination with rifampin for treatment of refractory chronic infections.

## Discussion

**Figure 7:**
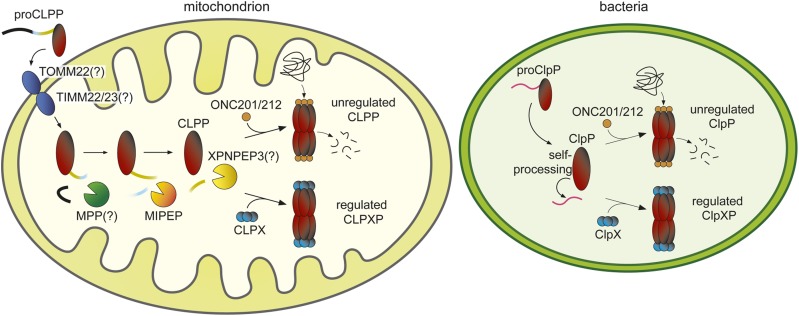
Schematic of CLPP processing and activation in human mitochondria *vs.* ClpP activation in bacteria.

Genetic interaction profiles can inform the mechanism of action of bioactive small molecules (Ho *et al.* 2011; Lin *et al.* 2019). Here, we used genome-wide CRISPR-based screens in a human cell line to systematically uncover genetic interactions of the imipridones ONC201 and ONC212. Loss of either CLPP and MIPEP conferred dramatic resistance to ONC201 and ONC212, and strongly suggested that these mitochondrial proteases mediate the toxic effects of the imipridones. Consistent with two recent studies that demonstrated direct activation of CLPP by these compounds (Graves *et al.* 2019; Ishizawa *et al.* 2019), we found that ONC201 and ONC212 activate CLPP *in vitro* and in a yeast surrogate genetic assay. Moreover, loss of MIPEP indirectly confers resistance because of its requirement in CLPP maturation (Figure 7). Ectopic activation of CLPP by ONC212 causes degradation of hundreds of proteins, not only in the mitochondrion but also in the cytoplasmic and nuclear compartments. Prompted by the similar mechanism of activation of human CLPP by the imipridones and ADEPs, we determined that the imipridones exhibit antimicrobial activity against all Gram-positive species tested, as well as some Gram-negative species. Commensurate with ADEP-activation of ClpP (Conlon *et al.* 2013), ONC212 treatment caused massive proteomic destruction in *S. aureus* with resultant potent bactericidal effects (Figure 7). Notably, ONC212 exhibited either additivity or synergism with a number of known antibiotics, and enhanced the ability of rifampin to eliminate antibiotic-tolerant persister cells in biofilms. These results have implications for imipridone mechanism of action, CLPP biogenesis, cancer cell resistance mechanisms, and antibiotic drug discovery.

### Genetic specificity of imipridone action

Our genome-wide screens appear to rule out a number of mechanisms of action ascribed to the imipridones (Allen *et al.* 2016). As we did not identify any strong resistance genes downstream of CLPP and MIPEP, it is unlikely that a single sensor or effector pathway propagates CLPP-induced damage to the rest of the cell. In particular, we did not identify known apoptotic effectors as strong resistance hits, as would have been expected for TRAIL-mediated death (Allen *et al.* 2013; Kline *et al.* 2016). Interestingly, our proteomic analysis revealed depletion of several cell cycle proteins, which may explain a previously described cell cycle arrest phenotype caused by ONC201 (Kline *et al.* 2016). We also did not recover HRI (EIF2AK1) or PKR (EIF2AK2) genes, or other mediators of the integrated stress response (Ishizawa *et al.* 2016; Kline *et al.* 2016), nor any dopaminergic receptors or other GPCR-related genes (Kline *et al.* 2018; Madhukar *et al.* 2019; Nii *et al.* 2019; Prabhu *et al.* 2019). Although it is possible that cell type-specific gene expression may explain the absence of these genetic hits, the most parsimonious explanation is that CLPP is the primary target of the imipridones. The proximal effect of unregulated CLPP activation on mitochondrial integrity is consistent with the established correlation between ONC201 sensitivity and reliance on mitochondrial respiration (Greer *et al.* 2018), as well as the depletion of proteins involved in oxidative phosphorylation (OXPHOS), as shown here and elsewhere (Greer *et al.* 2018; Graves *et al.* 2019; Ishizawa *et al.* 2019). The activation of CLPP by the imipridones represents a new avenue in the emerging anticancer strategy of targeting mitochondrial functions (Cole *et al.* 2015; Zong *et al.* 2016; Baccelli *et al.* 2019).

### Proteolytic processing of CLPP and resistance mechanisms

Protein import into the mitochondrion relies on a complex set of proteolytic events to remove targeting sequences and convert proteins into final mature forms (Gakh *et al.* 2002). Previously, by using an *E. coli*–human chimeric ClpP construct, it was suggested that the removal of the propeptide was due to self-processing (Kang *et al.* 2004), similar to bacterial ClpP maturation by self-processing (Maurizi *et al.* 1990). Our data in NALM-6 MIPEP population knockout cells and yeast *oct1*Δ cells show that MIPEP (or a MIPEP ortholog) is necessary for the generation of mature, catalytically competent CLPP. The requirement for MIPEP to process CLPP into a mature active form explains the genetic mechanism whereby MIPEP confers resistance to ONC201/212. As for many mitochondrial matrix proteins, processing by the mitochondrial processing peptidase (MPP) is likely first required to remove the import signal sequence, followed by subsequent cleavage of an octapeptide by MIPEP upon import into the mitochondrial matrix (Gakh *et al.* 2002). As the proCLPP sequence and the reported N-terminal amino acid of mature CLPP (Pro57) do not strictly adhere to the canonical octapeptide recognition sequence, an additional aminopeptidase, such as XPNPEP3, may mediate removal of the remaining Leu56 residue after MPP and MIPEP processing (Singh *et al.* 2017). The extensive processing steps needed for CLPP maturation into an active form all represent potential mechanisms for the acquisition of resistance to imipridones or other CLPP activators by cancer cells. XPNPEP3 and the MPP PMPCB subunit both scored in the top 350 resistance genes in the ONC201 screen but had no effect in the ONC212 screen (Table S1). Other mutations that indirectly compromise mitochondrial import or proteolytic processing functions may also confer partial resistance, as shown in our genetic screens. These bypass mechanisms will be counterbalanced by the relative dependence of each particular cancer on mitochondrial function (Zong *et al.* 2016; Greer *et al.* 2018).

### Imipridones as a new antibiotic scaffold

The unfolding antimicrobial resistance crisis is a global health and economic threat that demands new strategies for antibiotic development (Tyers and Wright 2019). Antibiotics currently used in the clinic comprise only a small number of chemically distinct scaffold classes, all of which are decades old and target a limited number of cellular processes (Brown and Wright 2016). Despite the potential of ADEPs as a new scaffold class with a novel mechanism of action, preclinical development of ADEPs as antibiotics has been hampered by synthetic tractability, chemical stability, and pharmacokinetic issues (Hinzen *et al.* 2006; Famulla *et al.* 2016; Li *et al.* 2017; Moreno-Cinos *et al.* 2019). The imipridones, which already have excellent pharmacodynamic and toxicological characteristics, offer an opportunity to revisit ClpP activators as antibacterials. ONC212, and to a lesser extent ONC201, can activate bacterial ClpP from both Gram-positive and -negative species, and exhibit good bactericidal activity against *S. aureus*, a major Gram-positive pathogen. Despite clear activation of recombinant *E. coli* ClpP *in vitro*, the imipridones have variable cellular activity against Gram-negative species due to the powerful innate resistance mechanisms in these organisms. Notably though, ONC212 inhibits growth of *N. gonorrhoeae*, for which highly antimicrobial-resistant strains are a serious public health threat (Costa-Lourenço *et al.* 2017). As some imipridone analogs can cross the blood–brain barrier (Allen *et al.* 2013, 2016), imipridone-based antimicrobials might hold promise in the treatment of bacterial meningitis, such as caused by *N. meningitidis* (Zouheir *et al.* 2019). Further refinement of the imipridone scaffold may improve its activity against Gram-negative species. Since ClpP is not essential in most bacterial species, imipridone resistance may arise through mutations that directly or indirectly cripple ClpP activity. However, because ClpP is often crucial for virulence, imipridone-resistant *clpP* mutant strains would likely be less pathogenic. Intriguingly, we found that ONC201, but not its more potent analog ONC212, modestly inhibits the growth of two mycobacterial species: *M. smegmatis* and an attenuated version of *M. tuberculosis*. We do not yet know if this cellular effect is due to activation of the heteromeric mycobacterial ClpP1P2 complex by ONC201 *vs.* inhibition of physiological ClpP function, as has been reported for ADEPs (Famulla *et al.* 2016). Regardless, this result raises the prospect that more selective imipridones might be developed to combat multidrug-resistant tuberculosis (TB), particularly in combination with rifampin and other cornerstone anti-TB drugs (Tiberi *et al.* 2018).

### Species-specific activators of ClpP

Drug-induced activation of either human CLPP or bacterial ClpP is a promising strategy for both anticancer (Bhandari *et al.* 2018; Wong *et al.* 2018; Wong and Houry 2019) and anti-infective drug discovery (Culp and Wright 2017; Bhandari *et al.* 2018). However, the potential cross-activation of human CLPP and bacterial ClpP by the current generation of imipridones raises the issue of unintended collateral effects. For example, imipridone-based anti-cancer therapy might adversely impact the microbiome or, conversely, the use of imipridones as antibiotics might negatively impact normal tissues that require high mitochondrial capacity. A number of compounds have been identified as ClpP activators, including the ADEPs and a variety of (semi)-synthetic analogs (Brötz-Oesterhelt *et al.* 2005; Carney *et al.* 2014b; Goodreid *et al.* 2016; Wong *et al.* 2018; Griffith *et al.* 2019), ADEP-based fragments (Carney *et al.* 2014a), the imipridones and the related TR series compounds (Allen *et al.* 2013; Graves *et al.* 2019; Ishizawa *et al.* 2019; Wong and Houry 2019), the natural product sclerotiamide (Lavey *et al.* 2016), and the synthetic compounds D9 (Stahl *et al.* 2018) and ACP1 (Leung *et al.* 2011). X-ray structures show that ONC201, ADEPs, and D9 all occupy the same binding site in human CLPP (Stahl *et al.* 2018; Wong *et al.* 2018; Ishizawa *et al.* 2019; Wong and Houry 2019). Although comparison of these human CLPP structures with several bacterial ClpP structures reveals that the binding pocket is highly conserved (Figure S5), divergent features of the pocket might nevertheless allow species-specific activators to be developed (Bhandari *et al.* 2018; Wong and Houry 2019). Indeed, D9 is only active against human CLPP (Stahl *et al.* 2018) and some ADEP analogs exert species-specific effects (Goodreid *et al.* 2016; Wong *et al.* 2018). Our data also suggest selectivity of ONC201 *vs.* ONC212 toward different bacterial and mycobacterial species. A structure-guided structure–activity relationship (SAR) campaign around the imipridone core could allow further elaboration of species-specific ClpP activators. For example, the variable Trp146 residue of human CLPP adjacent to the imipridone core is substituted by smaller residues in bacterial species (Wong *et al.* 2018) and may represent a potential point of discrimination (see Figure S5 and Supplemental Information for full discussion). Specific ClpP activators might thus be developed against a spectrum of pathogens including those not yet targeted by this approach, such as the malaria parasite *Plasmodium falciparum* (Florentin *et al.* 2017). Further diversification of the imipridone scaffold, and identification of new scaffolds that target ClpP, should allow the full therapeutic potential of the ClpP activation strategy to be realized.
